# Development of a comprehensive noninvasive prenatal test

**DOI:** 10.1590/1678-4685-GMB-2017-0177

**Published:** 2018-07-16

**Authors:** Carolina Malcher, Guilherme L. Yamamoto, Philip Burnham, Suzana A.M. Ezquina, Naila C.V. Lourenço, Sahilla Balkassmi, David S. Marco Antonio, Gabriella S.P. Hsia, Thomaz Gollop, Rita C. Pavanello, Marco Antonio Lopes, Egbert Bakker, Mayana Zatz, Débora Bertola, Iwijn De Vlaminck, Maria Rita Passos-Bueno

**Affiliations:** 1 Centro de Pesquisa sobre o Genoma Humano e Células-Tronco, Departamento de Genética e Biologia Evolutiva, Instituto de Biociências, Universidade de São Paulo, São Paulo, SP, Brazil; 2 Meinig School of Biomedical Engineering, Cornell University, Ithaca, NY, USA; 3 Faculdade de Medicina de Jundiaí, Jundiaí, SP, Brazil; 4 Department of Clinical Genetics, Laboratory for Diagnostic Genome Analysis (LDGA), Leiden University Medical Center, Leiden, The Netherlands; 5 Departamento de Obstetrícia e Ginecologia, Faculdade de Medicina, Universidade de São Paulo, São Paulo, SP, Brazil

**Keywords:** Cell-free DNA, next-generation sequencing, trisomy, noninvasive prenatal test, fetal fraction

## Abstract

Our aim was to develop and apply a comprehensive noninvasive prenatal test (NIPT) by using high-coverage targeted next-generation sequencing to estimate fetal fraction, determine fetal sex, and detect trisomy and monogenic disease without parental genotype information. We analyzed 45 pregnancies, 40 mock samples, and eight mother-child pairs to generate 35 simulated datasets. Fetal fraction (FF) was estimated based on analysis of the single nucleotide polymorphism (SNP) allele fraction distribution. A Z-score was calculated for trisomy of chromosome 21 (T21), and fetal sex detection. Monogenic disease detection was performed through variant analysis. Model validation was performed using the simulated datasets. The novel model to estimate FF was robust and accurate (r^2^= 0.994, *p*-value < 2.2e-16). For samples with FF > 0.04, T21 detection had 100% sensitivity (95% CI: 63.06 to 100%) and 98.53% specificity (95% CI: 92.08 to 99.96%). Fetal sex was determined with 100% accuracy. We later performed a proof of concept for monogenic disease diagnosis of 5/7 skeletal dysplasia cases. In conclusion, it is feasible to perform a comprehensive NIPT by using only data from high coverage targeted sequencing, which, in addition to detecting trisomies, also make it possible to identify pathogenic variants of the candidate genes for monogenic diseases.

## Introduction

The discovery of cell-free fetal DNA (cffDNA) in the maternal bloodstream (Lo *et al.*, 1997) has revolutionized prenatal diagnosis. Initially, cell-free DNA (cfDNA) was used for detecting qualitative traits, such as fetal sex (Lo *et al.*, 1998; Rijnders *et al.*, 2001; Wright *et al.*, 2012) and Rhesus D status (Faas *et al.*, 1998; Finning *et al.*, 2002). More recently, next-generation sequencing (NGS) technologies have provided the means for noninvasive detection of fetal aneuploidy with high sensitivity and specificity (Fan *et al.*, 2008; Ehrich *et al.*, 2011; Palomaki *et al.*, 2011; Sparks *et al.*, 2012a; Gil *et al.*, 2015; Norton *et al.*, 2015). Noninvasive prenatal testing (NIPT) using NGS of cfDNA is now being widely used as a screening test for the most common aneuploidies in the prenatal setting. Chromosome Y read count has been used for accurately determining fetal sex (Chiu *et al.*, 2011; Koumbaris *et al.*, 2016) and estimating fetal fraction (FF) (restricted for male fetuses only) (Fan *et al.*, 2008; Hudecova *et al.*, 2014; Xu *et al.*, 2016).

High coverage targeted sequencing allows the accurate detection of fetal alleles without requiring parental genotyping (Liao *et al.*, 2011). In addition to aneuploidy detection, this strategy enabled the identification of variants associated with monogenic diseases, especially *de novo* variants (Lam *et al.*, 2012; New *et al.*, 2014; Chitty *et al.*, 2015). This method also enabled the development of methods for FF estimation by using single nucleotide polymorphisms (SNPs) from sequencing analysis of maternal plasma cfDNA, thus avoiding the need of parental genotyping, and reducing laboratory steps and turnaround time (Jiang *et al.*, 2012; Sparks *et al.*, 2012b; Koumbaris *et al.*, 2016). FF estimation is crucial for test accuracy, because insufficient fetal cfDNA may lead to false negative results. Thus, measuring the presence of fetal DNA (independently of fetal sex) in maternal plasma in any test (e.g. trisomy detection) should improve its reliability. The aforementioned analyses are already being performed in clinical settings, although not within one single test. The development of parameters to perform all these analyses simultaneously by using only maternal plasma sequencing data may further reduce cost and turnaround time.

NIPT in Brazil is currently offered by private laboratories, and is performed by outsourcing the technology or the test itself. In the present report, we propose the implementation of an in-house NIPT by using high-coverage targeted NGS in order to estimate FF, determine fetal sex, and detect trisomy and monogenic disease without the need for parental genotypes. We used skeletal dysplasia (SD) as a monogenic disease model.

## Subjects and Methods

### Subjects and samples

Peripheral blood samples were collected from pregnant women (N=45) and non-pregnant individuals (8 mother and children pairs, N=16), the latter being obtained to establish a proof of concept of the test. Pregnant women were at least 18 years old, with singleton pregnancies, and at 10 to 36 gestational weeks. This study was approved by the Research Ethics Committee of Instituto de Biociências (Universidade de São Paulo - Brazil), and informed consent was obtained from all patients or legal tutors.

Blood samples were collected in EDTA tubes, and plasma processing took place within six hours. Blood samples were centrifuged at 1600 x *g* for 10 min, and recentrifuged at 16000 x *g* for another 10 min. Plasma cfDNA extraction of 2-4 mL was performed by using the QIAamp Circulating Nucleic Acid kit (Qiagen) following the manufacturer's protocol. cfDNA was first eluted in a total of 150 μL and then concentrated to 60 μL by vacuum centrifugation.

### Proof of concept – mock samples

To establish a proof of concept of the test and validate the bioinformatics pipeline, we generated 40 *in silico* pregnancy mock samples by mixing the fastq reads from both mothers and children (five different FFs for each one of the mother-child pairs). We mixed the fastq reads with different fractions in order to simulate different ‘fetal fractions’ for each pair, mimicking the progressive increase in FF during pregnancy from the first to the third trimester. Among these samples are pairs with children affected and unaffected by Down syndrome.

### High coverage next-generation targeted sequencing of plasma samples

For cfDNA library preparation, we used the NEBNext Ultra kit (New England Biolabs) according to the manufacturer's protocol. Libraries were indexed, multiplexed, captured for a gene panel using Nextera Rapid Capture (Illumina), and quantified by real-time quantitative PCR by using the KAPA Library Quantification kit (KAPA Biosystems). Libraries were then sequenced in a MiSeq system (Illumina) using the MiSeq Reagent kit v3 (2x75 cycles), as well as in a HiSeq system (Illumina) with a HiSeq Rapid SBS Kit v2 (2x100 cycles).

**Figure 1:**
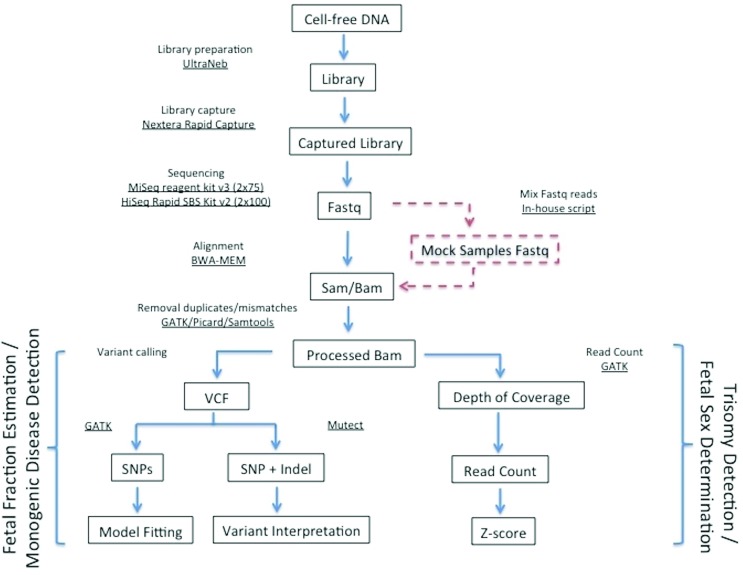
Test workflow. Red: performed only in the mother and child fastq files to generate mock samples.

The fastq files were aligned by BWA-MEM (Li and Durbin, 2010), duplicated reads were removed by Picard (http://broadinstitute.github.io/picard), realigned based on known local indels with GATK (McKenna *et al.*, 2010; DePristo *et al.*, 2011; Van der Auwera *et al.*, 2013), and reads with more than two mismatches were removed using Samtools (Li *et al.*, 2009). The mean coverage of BAM files was determined using Samtools Depth. For FF estimation, we performed variant call with all patients with GATK. For aneuploidy detection, we generated a Depth of Coverage file for each sample with GATK. For monogenic disease detection, the somatic variants were called by using Mutect (Cibulskis *et al.*, 2013). The workflow is outlined in Figure 1.

### Gene panel

For targeted sequencing, we used a panel of genes of clinical interest used in the routine diagnosis performed at HUG-CELL (Human Genome and Stem Cell Research Center). The panel consists of 497 genes of clinical interest (Table S1) belonging to the following groups of disorders: Hereditary Cancer, Skeletal Dysplasias/Craniofacial diseases, Neuromuscular/Neurodegenerative, Intellectual Deficiency/Autism, and Recessive Diseases Screening (http://genoma.ib.usp.br/pt-br).

We used SNPs to estimate FF, and read count to detect aneuploidy. To estimate FF, we used 6739 probes distributed in 388 genes across the autosomes (minus chromosomes 18 and 21, which were excluded because they are the most common trisomies and can affect minor allele fraction – MAF – estimate), comprising approximately 1.5 Mb. For the detection of trisomy on chromosome 21 (T21), variable regions were excluded to minimize variance in the read count analysis. These regions were frequently observed as false positive CNVs calls in Hidden Markov Model (HMM) analysis of NGS data in more than 100 patients, who were sequenced by the same panel for genetic diseases. After this correction, we used 240 probes distributed in 19 genes (approximately 33.5 Kb) across chromosome 21 for T21 detection.

### Fetal fraction estimation / Model fitting and evaluation

Maternal plasma had a mixture of cfDNA from the mother and fetus. For any biallelic SNP, there are four possible combinations of maternal/fetal genotypes. As *w*e do not know the genotype combination at each interrogated *locus a priori* (Table 1), we generated a model so as to fit our data and to estimate FF (developed with R, v. 3.2.3) based on each *locus* MAF (Supplementary Material Text 1).

**Table 1:** MAF given FF for different maternal/fetal genotype combinations in maternal plasma.

Possibilities	Origin	Genotype	Minor Allele	Mean of B allele fraction (MAF)
1	Maternal	AA	B	0
	Fetal	AA		
2	Maternal	AA	B	FF/2
	Fetal	AB		
3	Maternal	AB	B	0.5-FF/2
	Fetal	AA		
4	Maternal	AB	B	0.5
	Fetal	AB		

To evaluate the model and fitting accuracy, we used 2255 simulated samples (with different mean coverage and SNP number values), as well as 40 mock samples (from which we had the expected FF from fetal specific alleles), and 16 non-pregnant samples (either mother or child in which FF is expected to be 0).

### Detection of trisomy 21

To detect the frequency of T21, we used the read count generated by GATK Depth of Coverage. For each chromosome, we calculated the chromosome proportion defined as the sum of reads on that chromosome divided by the total reads of autosomes minus the chromosome of interest.

The reference dataset of each chromosome consisted of pregnant samples (including mock samples) with a fetus unaffected by trisomy. To normalize the reference dataset, we calculated the median and standard deviation for the reference dataset and removed the samples falling outside of three median absolute deviations. Importantly, the reference dataset has to contain samples sequenced with the same platform (MiSeq or HiSeq).

We then used a Z-score approach to calculate the genomic representation of the chromosome of interest compared to the reference dataset for each test sample:

Z-score_test sample_ = (P_test sample_ – P_mean reference samples_ / SD_mean reference samples_

(P = proportion of the chromosome of interest; SD = standard deviation)

### Fetal sex determination

For fetal sex determination, we used the chromosome Y read count. The reads covering the *SRY* gene were counted with GATK Depth of Coverage, and the proportion of chromosome Y was determined as the sum of reads of chromosome Y divided by total sum of autosome reads. The reference dataset consisted of female fetus pregnancies (including mock). It is important to note that the reference dataset has to contain samples sequenced with the same platform (MiSeq or HiSeq). Normalization was applied and a Z-score was calculated for T21 detection.

### Detection of monogenic disease and variant interpretation

Skeletal dysplasia (SD) is a group of bone and cartilage disorders that affect fetal development *in utero* or postnatally. Prenatal onset SDs are clinically detectable through gestational ultrasound presenting limb defects or reduction. Many of the prenatal onset SDs are autosomal dominant and lethal, but some of them are non-lethal. The molecular confirmation of the lethality of the fetus prior to birth would certainly help to manage the pregnancy.

The availability of probes for several genes of clinical interest in our panel (including several forms of SDs) allowed us to perform a specific analysis for this disease, with the aim of performing a proof of concept analysis in our data for the prenatal detection of monogenic diseases.

For the analysis of possible pathogenic variants, we performed variant call individually by Mutect (Cibulskis *et al.*, 2013), and annotated it by Annovar (Wang *et al.*, 2010) and several public databases (ExAC, Exome Variant Server, 1000 Genomes), including our in-house database of 609 Brazilian control exomes. We screened for rare variants (minor allele frequency < 0.5%) that are present only in genes related to dysplasia/craniofacial disorders (Table S2). For *de novo* variants in the fetus only, we expected to detect the MAF variant at approximately half of FF.

### Blind dataset for validation

For blind validation of our methodology, we used eight pregnant samples comprising controls and fetuses affected by T21 that were not previously known to test results. Library preparation and sequencing (by using HiSeq) were performed for the rest of the samples used in this work, as described above.

## Results

### Sample characterization and sequencing

A total of 61 peripheral blood samples were collected, 45 being from pregnant women and 16 from non-pregnant individuals (8 mothers and children pairs). The pregnant women were aged between 20 and 46 (mean: 32.5, SD ± 5.86) and at 10-36 gestational weeks (mean: 20.4, SD ± 9) (Table S3). Among the eight non-pregnant pairs used to generate the mock samples, we collected two children affected by Down syndrome (T21) and six unaffected by T21 (Table S4).

Sequencing of 47/61 samples was performed using MiSeq (33 pregnant women and 14 non-pregnant individuals), and yielded an average of 15.2 million raw reads per sample (ranging from 8,695,612 to 33,517,518). Mean coverage in BAM files was 191.65 X (39.99 X - 294.3 X, median: 208.6 X).

Sequencing of 14/61 samples by using HiSeq (12 pregnant women and two non-pregnant individuals) yielded an average of 106.9 million raw reads per sample (ranging from 35,316,152 to 246,692,132). Mean coverage in BAM files was 519.92X (201.6X – 928.38X, median: 522.75 X). Total average coverage (MiSeq and HiSeq altogether) comprised 267 X (Median: 222 X).

### Mock samples

Mock samples generated by a fastq file mixture instead of cfDNA mixture of mother and child have the advantage of allowing files with multiple FFs with low cfDNA input and cost, since it only requires sequencing mother and child once. However, there are intrinsic differences of PCR duplicates in the fastq files of both mother and child, which can be a confounding factor when estimating FF directly from the mock sample. In order to have an accurately expected FF for the mock samples, we employed two methods: fetal-specific alleles by using maternal genotype information (Chiu *et al.*, 2010; Liao *et al.*, 2012) (using only SNP positions with a coverage of at least 100X), and chromosome Y read count for male fetus pregnancies (Chiu *et al.*, 2011; Hudecova *et al.*, 2014). The estimated FFs in both approaches (fetal-specific alleles and chromosome Y read count) are strongly correlated, especially for high coverage samples (Pearson correlation r^2^ –all samples: 0.846, *p*=2.527e-06; coverage ³ 100x: 0.951, *p*=1.506e-08; coverage ³ 150X: 0.997, *p*=8.449e-07; coverage ³ 200X: 0.996, *p*=0.05119). Since the Y read count is only applicable to male fetus pregnancies, we used fetal-specific alleles to predict FF.

### Fetal fraction estimation

The fitting of the samples was performed, as explained in the Methods section, by comparing the MAF values distribution between the test sample and simulated samples for the specific MAF range (0.02–0.25) (Figure S1). A higher mean coverage and SNP number of simulated samples positively affect the model fitting, as expected (Figure S2).

We tested the model fitting accuracy for mean coverage and SNP number values obtained for our MiSeq sequenced samples (150X and 2000, respectively), which were lower than the samples sequenced by HiSeq. We found a high correlation between the expected and fitted FF values (Pearson correlation r^2^ =0.999, *p* < 2.2e-16), with median degree of deviation of 0.000 (-0.033–0.050), calculated as: (Expected-Fitted)/Expected (Figure S3).

**Figure 2:**
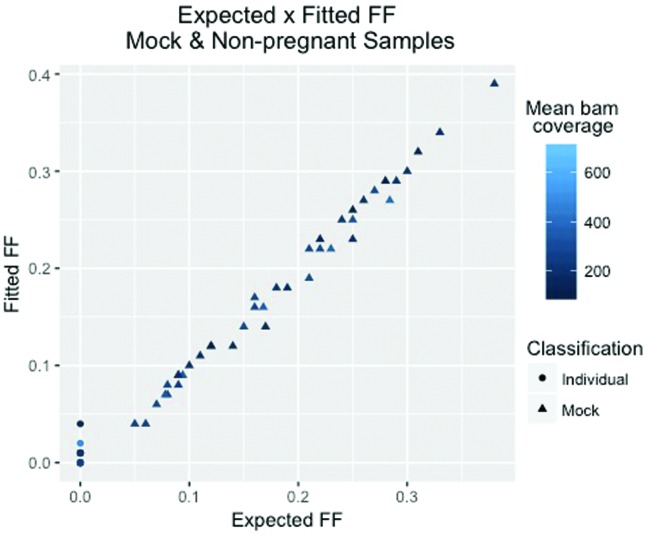
Evaluation of the modeled fetal fraction (FF) and the mean coverage effect. Individual: Non-pregnant sample. Shape and color incorporate both individual classification and mean coverage value, respectively.

The use of our mock and non-pregnant samples indicated that the model fitting is also accurate when using non-simulated samples (Pearson correlation r^2^=0.994, *p* < 2.2e-16) (Figure 2, Tables S4 and S5).

The developed model was then used to estimate FF for all samples (mock, pregnant, and non-pregnant). After VCF filtering, our samples had average SNP numbers of 4162 (11-5529, median: 4423) and 3990 (3514-4327, median: 4018) for MiSeq samples and HiSeq samples respectively (Tables S3, S4 and S5).

Mean fitted FF for pregnant samples was 0.12, varying between 0.02-0.30. Correlation analysis showed a strong positive correlation between FF and gestational age (Pearson correlation r ^2^ =0.5, *p*=4.4e-04). We did not find a significant association between FF and maternal weight (Pearson correlation r^2^ =-0.137; *p*=0.38) (Figure S4).

### Fetal sex determination

**Figure 3:**
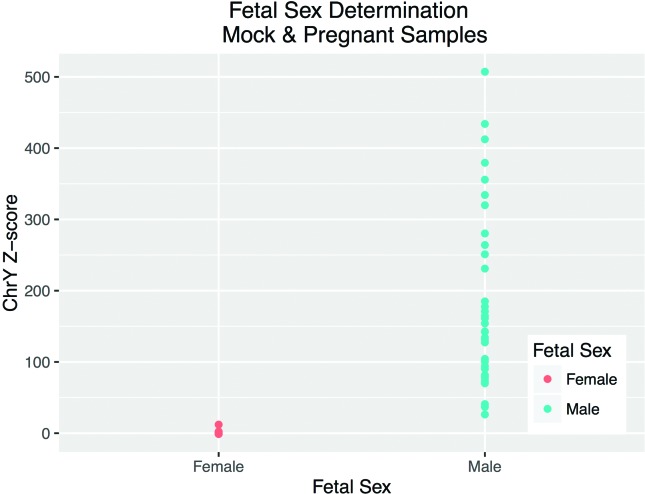
Fetal sex determination in 44 females and 37 males. Chromosome Y Z-score according to fetal sex.

For fetal sex determination, the normalized reference dataset consisted of 33 and 10 samples for MiSeq and HiSeq, respectively. Chromosome Y proportion and Z-score were calculated for each mock and pregnant sample (Tables S3 and S5). Male fetus pregnancies have an average proportion of 8.9e-05 (1.27e-05 – 2.38e-04) and Z-score of 185.35 (26.27 – 507.2), while female fetus pregnancies have an average proportion of 5.05e-07 (0 – 6.05e-06) and Z-score of 0.2 (-1.05 – 12.09). The groups do not overlap and, as such, they can be easily distinguished from one another. For the 81 samples for which we had confirmation of fetal sex (40/40 mock samples and 41/45 pregnant samples), we observed 100% accuracy (Figure 3), with 100% sensitivity (95% CI: 90.51%-100%), and 100% specificity (95% CI: 91.96%-100%).

#### Trisomy 21 detection

**Figure 4:**
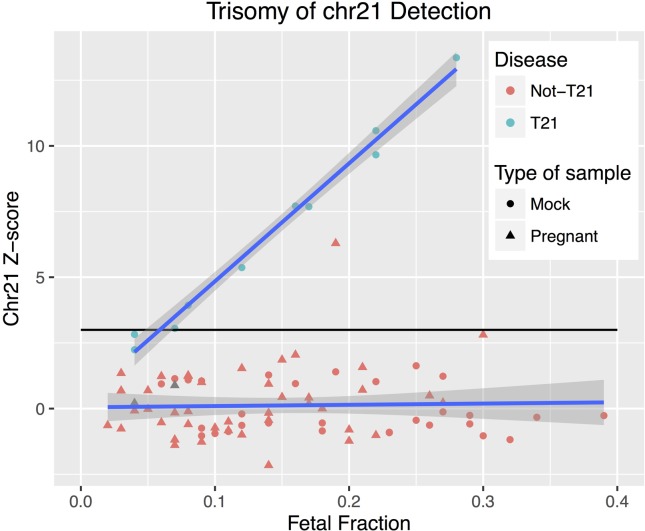
T21 detection. Chromosome 21 Z-score as function of FF. Color and shape incorporate both disease status (blue = T21, red = Not-T21 samples) and type of sample (circle = mock, triangle= samples with pregnant women), which represents 10 T21 samples (all mock), and 73 non-T21 samples (30 mock and 43 pregnant).

After normalization, the euploid reference dataset for T21 detection consisted of 54 and 16 samples for MiSeq and HiSeq, respectively. We then calculated chromosome 21 Z-score for all 83 samples (10 mock T21, 30 mock not-T21, and 43 non-affected pregnant samples) that had confirmation of the fetal diagnosis (two were excluded since they did not have confirmation), independently of FF. For the mock T21 samples, 8/10 had a positive Z-score (threshold 3.0) and 2/10 had a negative Z-score (false negatives) (Tables S3 and S5). The 43 non-affected pregnant samples had only one false positive (Z-score =6.29, FF=0.19) (Figure 4).

Using a threshold of FF0.04 as inclusion criteria, we had 76 out of the 83 samples (8 mock T21, 30 mock not-T21, and 38 pregnant samples) estimated with 100% sensitivity (95% CI: 63.06% to 100.00%) and 98.53% specificity (95% CI: 92.08% to 99.96%). By considering T21 frequency as 1:800 births, and the specificity and sensitivity values shown above, a false positive rate of 1.5% and a false negative rate of zero for our test were estimated.

We observed a positive correlation between FF and Z-score values for the T21 affected samples (Pearson correlation r^2^=0.994, *p*=6.013e-09), while this was not observed for unaffected samples (Pearson correlation r^2^=-0.033, *p*=0.7821).

#### Dataset blinded to diagnosis for validation

We also performed sequencing (by using HiSeq) and analysis of eight additional samples comprising affected (T21) and unaffected samples with pregnant women. The sequencing of these samples yielded an average of 16.5 million raw reads per sample (ranging from 10,338,602 – 23,619,856). Mean coverage in BAM files was 56.8X (32.7X – 83.5X, median: 57.6X).

The analysis of these samples revealed that five were from male fetuses and three from female fetuses. Regarding T21, five out of the eight samples were from T21 fetuses while three were from unaffected T21 fetuses. After disclosure of the original data, we verified 100% accuracy of our results regarding fetal sex determination and T21 detection (Table 2).

**Table 2:** Test results for blind dataset.

Sample	Trisomy	Test Result T21 (Z-score)	Fetus Gender	Test Result Fetal Sex (Z-score)
FD1500110	T21	T21 (12.9)	Male	Male (55.7)
FD1500068	T21	T21 (8.5)	Male	Male (27.41)
FD1500092	T21	T21 (7.8)	Male	Male (101.9)
FD1500073	T21	T21 (6.8)	Male	Male (51.44)
FD1500098	T21	T21 (4.4)	Female	Female (-1.05)
FD.15.00142	Not-T21	Not-T21 (0.2)	Female	Female (-1.05)
FD.15.00141	Not-T21	Not-T21 (0.17)	Female	Female (-1.05)
FD1500107	Not-T21	Not-T21 (-0.55)	Male	Male (98.8)

#### Monogenic disease detection – skeletal dysplasia

Seven samples from pregnant women had prenatal ultrasound findings suggesting SD. After VCF analysis, we detected known pathogenic variants in 5/7 samples (Table 3).

**Table 3:** Samples with women who were pregnant with fetuses diagnosed with SD by ultrasound

Patient	FF^1^	GW^2^	Gene	Pathogenic Mutation	AF^3^	Confirmed on child
F10177-2	0.22	32	*FLNB*	NM_001457:c.605T > C (p.M202T)	0.11	✓
F10775-1	0.12	29	*FGFR3*	NM_000142:c.742C > T (p.R248C)	0.07	✓
F10609-1	0.2	30	*FGFR3*	NM_000142:c.742C > T (p.R248C)	0.08	✓
F11247-1	0.13	26	*FGFR3*	NM_000142:c.742C > T (p.R248C)	0.034	✓
F11077-1	0.07	23	*FGFR3*	NM_000142: c.1108G > T(p.G370C)	0.031	NA
F10951-1	0.07	33	×	×	×	NA
F10774-1	0.11	33	×	×	×	NA

1 FF: fetal fraction.

2 GW: gestational weeks.

3 AF: allele fraction.

× Not found. NA: Not available.

We detected a pathogenic variant in sample F10177-2 located at the *FLNB* gene (NM_001457:c.605T > C:p.M202T), and associated with a lethal form of SD (Ateleosteogenesis type 1/Boomerang dysplasia) (Daniel *et al.*, 2012).

Pathogenic mutations in the *FGFR3* gene were identified in four patients. *FGFR3* mutations are associated with thanatophoric dysplasia (TD), an autosomal dominant disorder, which was the initial diagnostic hypothesis (DH) for the three samples in which we detected the mutation NM_000142:c.742C > T:p.R248C, the most common mutation associated with TD type I (Tavormina *et al.*, 1995; Wilcox *et al.*, 1998). The other sample harboring an *FGFR3* mutation (F11077-1) had an initial DH of campomelic dysplasia, with unspecific ultrasound findings (short bent bones, brachycephaly, and narrow thorax). This patient (F11077-1) had a rare mutation in *FGFR3* associated with TD type I as well (Rousseau *et al.*, 1996).

We were unable to detect pathogenic mutations in two samples (F10951-1 and F10774-1) that had an initial DH of osteogenesis imperfecta (OI). We did not have the child's genomic DNA to verify whether this was due to methodological reasons (unability to detect it noninvasively), or whether the mutation was not present in our panel. Although we did not find a pathogenic mutation for these cases, we did detect a VUS (variant of unknown significance) for patient F10774-1 (NM_001235.3:c.580C > A:p.R194S) located at *SERPINH1*, a gene already associated with a recessive form of OI (Bonafe *et al.*, 2015).

The MAF of these variants is about half of FF, as expected for *de novo* variants associated with autosomal dominant disorders. No other variant within the expected MAF was classified as pathogenic or probably pathogenic by using ACMG criteria (Richards *et al.*, 2015). Results were confirmed by sequencing the child's genomic DNA after birth, when available.

### Discussion

We have developed an NIPT for genetic diseases by using NGS that incorporates the following analysis: FF estimation (using only maternal plasma sequencing data), fetal sex determination, trisomy detection, and monogenic disease detection. A key strength of this study was the incorporation of all analyses in one single test, which was performed with the same gene panel used for the regular clinical genomic diagnosis in our center. This strategy requires only minimal modifications if the panel is up to date. To our knowledge, this is the first work to create a comprehensive test, and it has the advantage of allowing samples with different diagnostic purposes within the same laboratory workflow. While high coverage exome sequencing generally is not yet financially feasible for prenatal testing, this approach opens up the possibility to test hundreds of monogenic diseases with NGS, by targeting all coding sequences instead of solely relying on the investigation of mutational hot spots.

FF determination aids in avoiding false negative results and improves the detection of point mutations. Therefore, we developed a model to estimate FF that uses only plasma sequencing data regardless of fetal sex. Other groups use SNPs from targeted sequencing data to predict FF through a statistical binomial mixture model, relying on several mother-child genotype combinations to correctly predict FF (Jiang *et al.*, 2012; Sparks *et al.*, 2012b; Koumbaris *et al.*, 2016). We showed that it is possible to perform FF estimation with simpler statistics (MAF values vector comparison in R) by using only the most informative genotype combination (mother homozygous, child heterozygous).

Since FF is an important factor in NIPT accuracy and has been correlated with different maternal traits in other populations, we investigated its correlation with factors such as gestational age and maternal weight in Brazilian pregnant women. We found a positive correlation between FF and gestational age, in accordance with other reports (Lo *et al.*, 1998; Zimmermann *et al.*, 2012; Hudecova *et al.*, 2014; Rava *et al.*, 2014; Zhou *et al.*, 2015; Xu *et al.*, 2016). However, we did not find a significant correlation between FF and maternal weight as reported by others (Ashoor *et al.*, 2012; Wang *et al.*, 2013; Hudecova *et al.*, 2014). This lack of correlation may be attributable to our small sample size.

In this work, we established a threshold of 0.04 FF for T21 detection, as reported in the literature, for better accuracy. Literature data indicate high sensitivity and specificity for T21 detection by NGS varying between 94.4% - 100% and 97.95% - 100%, respectively (Chiu *et al.*, 2011; Gil *et al.*, 2015), and our test sensitivity and specificity values were within these ranges, showing that we have high sensitivity and specificity for T21 detection. We also performed the test in a blind dataset for validation, resulting in 100% accuracy for fetal sex and T21 determination.

The two false negative samples for T21 detection have fitted FFs of 0.04, which is the detection limit found in the literature (Ehrich *et al.*, 2011; Norton *et al.*, 2012; Palomaki *et al.*, 2012; Sparks *et al.*, 2012b), so they were expected to present low Z-scores (Ehrich *et al.*, 2011; Norton *et al.*, 2012; Palomaki *et al.*, 2012; Sparks *et al.*, 2012b).

The one false positive result for T21 observed in our sample can be due to several factors: confined placental mosaicism, fetal mosaicism, vanishing twin, or even maternal malignancies (Osborne *et al.*, 2013; Grati *et al.*, 2014; Bianchi *et al.*, 2015). We had a high positive correlation for the T21-affected samples, and the false positive sample did not fall onto the correlation line, as reported by others (Hudecova *et al.*, 2014). Corroborating the estimated high sensitivity and specificity of our test, we verified that the NIPT test in one of the pregnant women (F10117-1), who was referred to us with a positive diagnosis for T21 from a different clinical service, was negative for T21. The follow up of this case revealed that the child was born normal, in accordance with our NIPT screening test.

As shown previously, it is possible to perform fetal sex determination with targeted NGS (Chiu *et al.*, 2011; Koumbaris *et al.*, 2016). In this work, we demonstrated that it is possible to determine fetal sex with 100% accuracy by only one probe on chromosome Y instead of multiple probes (Koumbaris *et al.*, 2016).

Monogenic disease testing for SD was performed on seven cases, with a detection rate of 71% (5/7), thus demonstrating our test's capacity to incorporate detection of monogenic diseases, especially in *de novo* or paternally inherited variants. By using children's genomic DNA (unpublished data from our center), our noninvasive SD detection rate was similar to the postnatal detection rate (75/125 = 60%) by using child's genomic DNA (unpublished data from our center).

Thanatophoric, achondroplasia, and osteogenesis imperfecta are among the most common types of SD (Unger *et al.*, 1993; Milks *et al.*, 2017). For patient F11077-1, who had an initial DH of campomelic dysplasia, we confirmed the diagnosis as TD type I. Despite the existence of clinical overlap, this differential diagnosis is important because TD is lethal, while campomelic dysplasia is not always lethal. This differential diagnosis is also important for the medical team in the postpartum management as well as for the family's psychological preparation.

In the two patients for whom pathogenic mutations were not detected (F10951-1 and F10774-1), the DH was OI. For these patients, we cannot discount the hypothesis of the pathogenic mutation being in a gene absent from our panel, since the number of genes associated with SD has grown at a fast pace in recent years, especially due to the advent of NGS (Bonafe *et al.*, 2015). Another possible explanation is that the mutation could reside in an intronic region, or it could be a deletion, which would be overlooked by the currently available tools. For patient F10774-1, we detected a VUS in a gene that is already associated with a recessive form of OI. It is possible that this patient had a recessive form of the disease, and the lack of identification of the second mutation may be a limitation in identifying mutations that are present in the mother.

Chitty *et al.* (2015) have recently demonstrated the effectiveness of NIPT to detect *FGFR3*-related SDs. However, they targeted hotspots in the gene, which has the pitfall of possibly overlooking pathogenic variants. Dan *et al.* (2016) showed the feasibility of using targeted sequencing for 16 genes. However, they used maternal and paternal genomic DNA sequencing for variant detection. Comparatively, our test might be more advantageous because we are covering the entire coding sequence of hundreds of genes associated with monogenic disorders (therefore covering all exonic variants and many differential diagnoses), and also because we are able to perform the detection by using only plasma sequencing, thus lowering costs and turnaround time. These results suggest the possibility to expand our approach for detecting other monogenic dominant diseases, particularly those caused by *de novo* or paternally inherited variants. NIPT as a screening test for dominant disorders should be considered in the near future, particularly with the increase of the reproductive age in most populations together with the burden of *de novo* paternal mutations with aging, and the effect of selfish mutations in paternal gonads (Maher *et al.*, 2016).

It is important to note that for the five SD cases in which we detected the pathogenic mutation, the AF is about half of the FF, which is expected for autosomal dominant disorders. This also demonstrates that our FF estimation model is accurate and helpful for detecting pathogenic mutations, since we can target the mutation within the expected AF according to the disease inheritance model.

We showed the relevance of using targeted sequencing to develop an integrated NIPT (using only maternal plasma) by combining all analyses (fetal fraction estimation, fetal sex determination, trisomy, and monogenic disease detection). Further reduction of sequencing costs will enable an even higher coverage, thus improving the ability to detect autosomal recessive or X-linked mutations more accurately, when the mother is heterozygous for the variant.

To our knowledge, we are the first group in Brazil to develop an in-house, non-invasive prenatal test performed with NGS. NIPT is presently available for patients in Brazil, but the test is either performed abroad or through outsourcing technology. In this work, we demonstrated that it is indeed possible to perform NIPT for several fetal diseases by using only plasma sequencing data, a practicable amount of targeted sequencing, and relatively simple statistics.

## Supplementary material

The following online material is available for this article:

Figure S1 Fit procedure for FF estimation.

Figure S2 Evaluation of the model according to different fetal fractions (FF).

Figure S3 Evaluation of the fitting for mean coverage value = 150X and SNP numbers = 2000.

Figure S4 Correlations between gestational week, fetal fraction and maternal weight.

Table S1 Genes present in the clinical gene panel.

Table S2 Genes for dysplasia/craniofacial disorders present in the clinical panel.

Table S3 Summary of pregnant samples.

Table S4 Summary of non-pregnant individuals.

Table S5 Summary of mock samples.

Supplementary Text 1
