# Supplementary material for: Development of a comprehensive noninvasive prenatal test
Source: Genet Mol Biol. 2018 Jul 16;41(3):545–54. doi: 10.1590/1678-4685-GMB-2017-0177 (PMC6136382; doi:10.1590/1678-4685-GMB-2017-0177)
Supplement: Supplementary file 1 [file 1415-4757-GMB-1678-4685-GMB-2017-0177-suppl5.pdf]

Supplementary Material to "Development of a comprehensive noninvasive prenatal test"

Table S1 - Genes present on the clinical gene panel.

Genes									
AARS	*CACNA1S*	*CRYAA*	*FANCF*	*GNAI3*	*MAP2K1*	*NBN*	*POLR1D*	*SALL1*	*TCF12*
ABCD1	*CANT1*	*CRYAB*	*FANCG*	*GNAS*	*MATN3*	*NDN*	*POMGNT1*	*SATB2*	*TCF4*
ACADM	*CAPN3*	*CTDP1*	*FBLN5*	*GRHL3*	*MBD2*	*NDRG1*	*POMT1*	*SBDS*	*TCOF1*
ACADVL	*CAV3*	*CTNNB1*	*FBN1*	*GRIK1*	*MBD5*	*NDUFV2*	*POMT2*	*SBF2*	*TERT*
ACAT1	*CBS*	*CTSK*	*FECH*	*HADHA*	*MC2R*	*NEFL*	*POR*	*SCN4A*	*TFAP2A*
ACP5	*CCDC26*	*CUBN*	*FGD4*	*HADHB*	*MCCC1*	*NF1*	*POTED*	*SCO2*	*TG*
ACTA1	*CD3D*	*CUL7*	*FGF8*	*HBB*	*MCCC2*	*NIPA1*	*PPIB*	*SEPN1*	*TGFBR1*
ACTB	*CD3E*	*CYP21A2*	*FGFR1*	*HERC2*	*MECP2*	*NIPBL*	*PQBP1*	*SERPINH1*	*TGFBR2*
ACTG1	*CDH1*	*CYP27A1*	*FGFR2*	*HES7*	*MED25*	*NKX3-2*	*PRKAR1A*	*SETBP1*	*TGIF1*
ADA	*CDH2*	*CYP7B1*	*FGFR3*	*HLCS*	*MEGF10*	*NOG*	*PRPS1*	*SETX*	*TH*
ADAMTS18	*CDH23*	*DAG1*	*FHL1*	*HMGCL*	*MEN1*	*NOL8*	*PRSS1*	*SGCA*	*TMC1*
ADAMTSL2	*CDH7*	*DBT*	*FIG4*	*HNRPDL*	*MESP2*	*NOTCH3*	*PRX*	*SGCB*	*TMIE*
ADCK3	*CDH8*	*DCLRE1C*	*FILIP1L*	*HRAS*	*MFN2*	*NPC1*	*PSAT1*	*SGCD*	*TMPRSS15*
AFG3L2	*CDK4*	*DDR2*	*FKBP10*	*HSPB1*	*MID1*	*NPC2*	*PSPH*	*SGCG*	*TMPRSS3*
ALDH3A2	*CDKL5*	*DES*	*FKBP14*	*HSPB8*	*MIR1205*	*NPR2*	*PTCH1*	*SH3BP2*	*TNFRSF11A*
ALDH7A1	*CDKN2A*	*DFNB31*	*FKRP*	*IDUA*	*MIR1206*	*NRAS*	*PTEN*	*SHANK3*	*TP53*

**Genes**									
ALPL	*CFL1*	*DHCR24*	*FKTN*	*IFT80*	*MIR1207*	*NSD1*	*PTH1R*	*SHH*	*TP63*
ALX1	*CFTR*	*DHCR7*	*FLNA*	*IL11RA*	*MIR1208*	*NT5C3*	*PTPN11*	*SHOC2*	*TPM2*
ALX3	*CHEK2*	*DLAT*	*FLNB*	*IL2RG*	*MIR140*	*NTNG1*	*PTS*	*SHOX*	*TPM3*
ALX4	*CHST14*	*DLL3*	*FLNC*	*IL7R*	*MIR200B*	*OFD1*	*PVRL1*	*SIX3*	*TPO*
AMN	*CHST3*	*DMD*	*FMR1*	*IRF6*	*MLH1*	*OGN*	*PVT1*	*SLC17A6*	*TRAPPC2*
ANO5	*CLCN1*	*DNAJB6*	*FOLR1*	*ITGA7*	*MMAA*	*OTC*	*PYGM*	*SLC19A3*	*TRIM32*
APC	*CLDN14*	*DNM2*	*FOXE1*	*IVD*	*MMAB*	*OTOF*	*QDPR*	*SLC22A5*	*TRIP11*
APTX	*CLPTM1L*	*DUOX2*	*FOXG1*	*JAG1*	*MMACHC*	*OXCT1*	*RAB23*	*SLC25A13*	*TRPC6*
ARG1	*CLRN1*	*DYM*	*FOXP2*	*JAK3*	*MMADHC*	*PABPN1*	*RAB7A*	*SLC25A15*	*TRPS1*
ARHGAP29	*CNTNAP2*	*DYNC2H1*	*FTCD*	*JAM2*	*MMP13*	*PAFAH1B1*	*RABEP2*	*SLC26A2*	*TRPV4*
ARX	*COL10A1*	*DYRK1A*	*FUS*	*KATNAL2*	*MMP9*	*PAH*	*RAD50*	*SLC26A4*	*TSC1*
ASL	*COL11A1*	*DYSF*	*FXN*	*KBTBD13*	*MOCS1*	*PALB2*	*RAD51*	*SLC2A1*	*TSC2*
ASPA	*COL11A2*	*EDN1*	*GADD45G*	*KCNE1*	*MOCS2*	*PAPSS2*	*RAD51C*	*SLC5A5*	*TSHB*
ASS1	*COL18A1*	*EFNB1*	*GALNS*	*KIAA1267*	*MPZ*	*PAX8*	*RAD51D*	*SMAD4*	*TSHR*
ATL1	*COL1A1*	*EGR2*	*GALNT1*	*KIF22*	*MRAP*	*PCBD1*	*RAD51L3*	*SMARCAL1*	*TSHZ1*
ATM	*COL1A2*	*EIF2AK3*	*GALNT12*	*KIF5A*	*MRE11A*	*PCCA*	*RAF1*	*SNAP29*	*TSPEAR*
ATP7A	*COL2A1*	*EIF4A3*	*GALT*	*KRAS*	*MSH2*	*PCCB*	*RAG1*	*SNRPN*	*TWIST1*
ATP7B	*COL3A1*	*ELN*	*GAMT*	*L1CAM*	*MSH3*	*PCDH15*	*RAG2*	*SOD1*	*UBE3A*
ATR	*COL5A1*	*EMD*	*GARS*	*LAMA2*	*MSH6*	*PDGFRA*	*RAI1*	*SOS1*	*USH1C*

**Genes**									
AXIN2	*COL5A2*	*EPCAM*	*GATM*	*LAMP2*	*MSTN*	*PDHA1*	*RARA*	*SOS2*	*USH1G*
B4GALT6	*COL6A1*	*EPHA3*	*GCDH*	*LARGE*	*MSX1*	*PDHX*	*RBBP8*	*SOST*	*USH2A*
BAP1	*COL6A2*	*ERF*	*GCH1*	*LEMD3*	*MSX2*	*PDSS1*	*RECQL4*	*SOX9*	*USH2A*
BARD1	*COL6A3*	*ERG*	*GDAP1*	*LEPRE1*	*MTHFR*	*PDSS2*	*REEP1*	*SP7*	*USHBP1*
BCKDHA	*COL9A1*	*ETHE1*	*GDF5*	*LFNG*	*MTM1*	*PFKL*	*RET*	*SPAST*	*USP25*
BCKDHB	*COL9A2*	*EVC*	*GDF6*	*LIFR*	*MTMR2*	*PHF8*	*RGMA*	*SPG11*	*VAPB*
BIN1	*COL9A3*	*EVC2*	*GIF*	*LIPI*	*MTR*	*PHGDH*	*RHD*	*SPG7*	*VHL*
BMP4	*COMP*	*EXO1*	*GJA1*	*LITAF*	*MTRR*	*PLCB4*	*RIT1*	*SPR*	*WNT3*
BMPR1A	*COQ2*	*EXT1*	*GJB1*	*LMAN1*	*MUT*	*PLEC*	*RMRP*	*SRY*	*XRCC3*
BRAF	*COQ9*	*FAF1*	*GJB2*	*LMBRD1*	*MUTYH*	*PLOD1*	*ROR2*	*STK11*	*ZAP70*
BRCA1	*CP*	*FAH*	*GJB3*	*LMNA*	*MYC*	*PMP22*	*RPS27L*	*SUMO1*	*ZIC2*
BRCA2	*CPS1*	*FANCA*	*GJB6*	*LRP5*	*MYO6*	*PMS1*	*RTTN*	*TARDBP*	
BRIP1	*CREBBP*	*FANCC*	*GLB1*	*LRSAM1*	*MYO7A*	*PMS2*	*RUNX1*	*TBX1*	
BTD	*CRISPLD2*	*FANCD2*	*GLI2*	*LTBP4*	*MYOT*	*POGZ*	*RUNX2*	*TBX22*	
C21orf29	*CRTAP*	*FANCE*	*GLI3*	*LZTR1*	*NAGS*	*POLR1C*	*RYR1*	*TCAP*	
