# Supplementary material for: Development of a comprehensive noninvasive prenatal test
Source: Genet Mol Biol. 2018 Jul 16;41(3):545–54. doi: 10.1590/1678-4685-GMB-2017-0177 (PMC6136382; doi:10.1590/1678-4685-GMB-2017-0177)
Supplement: Supplementary file 2 [file 1415-4757-GMB-1678-4685-GMB-2017-0177-suppl10.pdf]

Supplementary Material to "Development of a comprehensive noninvasive prenatal test"

Supplementary Text 1

We generated a model to fit our data and estimate FF based upon MAF information (developed with R, v. 3.2.3). The model takes into account only biallelic *loci*, incorporates an error rate due to the finite sampling, and assumes an *a priori* maternal/fetal genotype combination frequency in maternal plasma (as seen in our own data and described by Jiang *et al.* (2012).

- Homozygous/homozygous: 0.7
- Homozygous/heterozygous: 0.1
- Heterozygous/homozygous: 0.1
- Heterozygous/heterozygous: 0.1

The model generates simulated samples based upon characteristics of the test sample (SNP number and coverage) for a range of different FF values (0 to 0.4, by 0.01). Simulations are then performed for each one of the tested FF values, and the distribution of the MAF values (range: 0.02-0.25, corresponding to the most informative peak - mother homozygous, child heterozygous) is evaluated for the test sample and compared to the same range for each one of the different FF simulated samples. Fitting is then performed based on similarity between simulated and test sample (lowest difference between MAF distributions). Fitting can be performed multiple times and have the mean and standard deviation calculated.

For FF estimation using SNPs we filtered the vcf by:

- Only SNPs on autosomes other than chromosomes 18 and 21 (although we did not perform trisomy 18 detection because of lack of samples, we already set our pipeline to do so)
- Excluded LowQual SNPs
- Base coverage>= 100 X
