# Supplementary material for: Development of a comprehensive noninvasive prenatal test
Source: Genet Mol Biol. 2018 Jul 16;41(3):545–54. doi: 10.1590/1678-4685-GMB-2017-0177 (PMC6136382; doi:10.1590/1678-4685-GMB-2017-0177)
Supplement: Supplementary file 3 [file 1415-4757-GMB-1678-4685-GMB-2017-0177-suppl6.pdf]

Supplementary Material to "Development of a comprehensive noninvasive prenatal test"

Table S2 - Genes for dysplasia/craniofacial disorders present in the clinical panel.

Genes									
ACP5	*COL18A1*	*DLL3*	*FGFR1*	*GNAI3*	*LRP5*	*PLS3*	*SATB2*	*TBX22*	*TSHZ1*
ADAMTS18	*COL1A1*	*DYM*	*FGFR2*	*GNAS*	*MATN3*	*POLR1C*	*SBDS*	*TBX6*	*TWIST1*
ADAMTSL2	*COL1A2*	*DYNC2H1*	*FGFR3*	*GRHL3*	*MESP2*	*POLR1D*	*SERPINF1*	*TCF12*	*WNT1*
ALPL	*COL2A1*	*EDN1*	*FIG4*	*HES7*	*MMP13*	*POR*	*SERPINH1*	*TCOF1*	*WNT3*
ALX1	*COL3A1*	*EFNB1*	*FKBP10*	*HUWE1*	*MMP9*	*PPIB*	*SH3BP2*	*TFAP2A*	*ZIC1*
ALX3	*COL5A1*	*EIF2AK3*	*FKBP14*	*IFITM5*	*MSX1*	*PRKAR1A*	*SHH*	*TGFBR1*	*ZIC2*
ALX4	*COL5A2*	*EIF4A3*	*FLNA*	*IFT80*	*MSX2*	*PTCH1*	*SHOX*	*TGFBR2*	
ANO5	*COL9A1*	*ELN*	*FLNB*	*IL11RA*	*NKX3-2*	*PTH1R*	*SIX3*	*TGIF1*	
BMP1	*COL9A2*	*ERF*	*GALNS*	*IRF6*	*NOG*	*PVRL1*	*SLC26A2*	*TMEM38B*	
CANT1	*COL9A3*	*EVC*	*GDF5*	*KIF22*	*NPR2*	*RAB23*	*SMARCAL1*	*TNFRSF11A*	
CHST14	*COMP*	*EVC2*	*GDF6*	*LEMD3*	*OFD1*	*RECQL4*	*SOST*	*TP63*	
CHST3	*CREB3L1*	*EXT1*	*GJA1*	*LEPRE1*	*PAPSS2*	*RMRP*	*SOX9*	*TRAPPC2*	
COL10A1	*CRTAP*	*FBLN5*	*GLB1*	*LFNG*	*PLCB4*	*ROR2*	*SP7*	*TRIP11*	
COL11A1	*CTSK*	*FBN1*	*GLI2*	*LIFR*	*PLOD1*	*RUNX2*	*SPARC*	*TRPS1*	
COL11A2	*DDR2*	*FGF8*	*GLI3*	*LMNA*	*PLOD2*	*SALL1*	*TBX1*	*TRPV4*	
