# Supplementary material for: Development of a comprehensive noninvasive prenatal test
Source: Genet Mol Biol. 2018 Jul 16;41(3):545–54. doi: 10.1590/1678-4685-GMB-2017-0177 (PMC6136382; doi:10.1590/1678-4685-GMB-2017-0177)
Supplement: Supplementary file 4 [file 1415-4757-GMB-1678-4685-GMB-2017-0177-suppl7.pdf]

Supplementary Material to "Development of a comprehensive noninvasive prenatal test"

Table S3 - Summary of pregnant samples.

Sample	Trisomy	Seq[1]	Fetal sex[2]	Maternal age (years)	GW[3]	Mean bam coverage (X)	Mean SNP number	Fitted FF	Stdev FF	ChrY prop	ChrY Z-score	Chr21 prop	Chr21 Z-score
P2183	Not-T21	HiSeq	Male	33	19	357.4	3974	0.14	0.006	5.57E-05	90.48	0.033770	-0.166
P2182	Not-T21	MiSeq	Male	35	27	208.6	4968	0.17	0.006	7.75E-05	164.50	0.034113	0.414
F9940-1	Not-T21	MiSeq	Male	37	11.5	141.2	3757	0.17	0.010	6.33E-05	134.21	0.034034	0.181
F9914-1	Not-T21	MiSeq	Female	35	13	219.1	4813	0.12	0.006	7.11E-07	0.70	0.034490	1.533
F9892-1	Not-T21	MiSeq	Male	35	12	244.2	5239	0.06	0.012	3.32E-05	70.01	0.034388	1.230
F9891-1	Not-T21	MiSeq	Male	32	20	269.7	4620	0.03	0.006	4.45E-05	94.11	0.033715	-0.766
F9614-1	Not-T21	MiSeq	Female	32	12	39.99	11	0.08	0.040	0	-0.81	0.033771	-0.600
F9531-1	Not-T21	MiSeq	Male	36	20	97.65	2999	0.05	0.006	1.77E-05	36.94	0.034206	0.689
F9530-1	Not-T21	MiSeq	Female	37	11	226.9	4213	0.02	0.006	5.78E-07	0.42	0.033758	-0.638
F9269-1	Not-T21	MiSeq	Male	46	10	186.9	3862	0.05	0.021	3.48E-	73.42	0.03396	-0.019

Sample	Trisomy	Seq[1]	Fetal sex[2]	Maternal age (years)	GW[3]	Mean bam coverage (X)	Mean SNP number	Fitted FF	Stdev FF	ChrY prop	ChrY Z-score	Chr21 prop	Chr21 Z-score
										05		7	
F9198-1	Not-T21	MiSeq	Female	34	17.5	222	4404	0.08	0.006	1.37E-06	2.11	0.033937	-0.108
F9176-1	Not-T21	MiSeq	Female	30	11.5	173.6	3875	0.14	0.000	7.83E-07	0.86	0.033825	-0.438
F9151-1	Not-T21	MiSeq	Female	35	31	80.04	1906	0.1	0.015	0	-0.81	0.033727	-0.728
F9051-1	Not-T21	MiSeq	Female	35	11	271.6	4584	0.15	0.006	0	-0.81	0.034120	0.435
F8998-1	Not-T21	MiSeq	Male	21	26	161.1	3707	0.14	0.006	6.20E-05	131.44	0.033245	-2.158
F8850-1	Not-T21	MiSeq	Female	44	11	203.9	4222	0.11	0.000	0	-0.81	0.033693	-0.831
F8775-1	Not-T21	MiSeq	NA	37	18	62.71	234	0.09	0.045	2.53E-05	53.15	0.033545	-1.267
F8762-1	Not-T21	MiSeq	Female	38	10	147.2	3373	0.09	0.012	0	-0.81	0.034311	1.002
F8660-1	Not-T21	MiSeq	Female	35	13	294.3	4647	0.03	0.006	5.41E-07	0.34	0.034427	1.347
F8659-1	Not-T21	MiSeq	Female	36	12	233.1	4375	0.03	0.000	6.05E-06	12.09	0.034203	0.683
F8642-1	Not-T21	MiSeq	Female	39	11	162.1	5355	0.06	0.010	0	-0.81	0.033795	-0.529

Sample	Trisomy	Seq[1]	Fetal sex[2]	Maternal age (years)	GW[3]	Mean bam coverage (X)	Mean SNP number	Fitted FF	Stdev FF	ChrY prop	ChrY Z-score	Chr21 prop	Chr21 Z-score
F8626-1	Not-T21	MiSeq	NA	27	12	132.2	4590	0.26	0.006	6.06E-05	128.45	0.034139	0.492
F8614-1	Not-T21	MiSeq	Male	29	13	66.12	362	0.04	0.010	1.27E-05	26.28	0.033946	-0.079
F11247-1	Not-T21	HiSeq	Female	30	26	620.6	4188	0.13	0.006	2.42E-07	-0.66	0.033613	-0.803
F11091-1	NA	HiSeq	NA	35	15	928.38	4327	0.04	0.010	2.47E-05	39.53	0.033861	0.208
F11077-1	Not-T21	HiSeq	Female	20	23	641.7	4201	0.07	0.006	1.11E-06	0.77	0.033519	-1.187
F10951-1	Not-T21	HiSeq	Female	23	33.5	374.7	4070	0.07	0.000	2.09E-06	2.38	0.033469	-1.392
F10855-1	Not-T21	HiSeq	Male	36	23	418.3	4018	0.21	0.000	9.97E-05	162.78	0.034197	1.573
F10795-1	Not-T21	HiSeq	Female	31	12	541.4	4038	0.08	0.000	2.47E-07	-0.65	0.034120	1.262
F10775-1	Not-T21	HiSeq	Male	27	29	356.5	4009	0.12	0.000	4.92E-05	79.80	0.033566	-0.996
F10774-1	Not-T21	HiSeq	Male	22	33	592	4235	0.11	0.006	4.80E-05	77.82	0.033688	-0.500
F10764-1	Not-T21	HiSeq	Female	26	26	463.8	4002	0.07	0.000	4.23E-07	-0.36	0.033773	-0.154
F10609-1	Not-T21	HiSeq	NA	34	30	556.3	4134	0.2	0.006	1.13E-	0.80	0.03350	-1.231

Sample	Trisomy	Seq[1]	Fetal sex[2]	Maternal age (years)	GW[3]	Mean bam coverage (X)	Mean SNP number	Fitted FF	Stdev FF	ChrY prop	ChrY Z-score	Chr21 prop	Chr21 Z-score
										06		8	
F10396-1	Not-T21	MiSeq	Male	40	36	267.9	4656	0.19	0.162	0.00017827	379.45	0.036096	6.292
F10351-1	Not-T21	MiSeq	Female	32	33	283.3	5166	0.3	0.012	2.83E-07	-0.21	0.034921	2.810
F10177-2	Not-T21	MiSeq	Female	20	32	194.2	4814	0.22	0.010	8.26E-07	0.95	0.033631	-1.014
F10157-1	Not-T21	MiSeq	Female	39	29	242.4	5066	0.16	0.010	0	-0.81	0.034663	2.046
F10138-1	Not-T21	MiSeq	Male	33	25	210.2	4188	0.18	0.006	0.00010868	231.01	0.033973	0.001
F10117-1	Not-T21	MiSeq	Female	25	30	233.6	4356	0.21	0.000	6.19E-07	0.51	0.034212	0.710
F10113-1	Not-T21	MiSeq	Male	34	35	212.5	4898	0.27	0.006	0.00013179	280.30	0.034049	0.226
F10063-1	Not-T21	MiSeq	Female	30	19.5	253.2	5258	0.08	0.000	6.29E-07	0.53	0.034390	1.237
C26729	Not-T21	MiSeq	Male	28	20	105.6	2176	0.07	0.000	4.93E-05	104.35	0.033574	-1.183
C26139	Not-T21	MiSeq	Female	35	11	250.2	5085	0.15	0.010	6.33E-07	0.54	0.034600	1.857
C24778	NA	HiSeq	Male	33	8	201.6	3514	0.07	0.000	2.55E-05	40.85	0.034027	0.884

Sample	Trisomy	Seq[1]	Fetal sex[2]	Maternal age (years)	GW[3]	Mean bam coverage (X)	Mean SNP number	Fitted FF	Stdev FF	ChrY prop	ChrY Z-score	Chr21 prop	Chr21 Z-score
C18177	Not-T21	MiSeq	Female	25	36	269.9	4367	0.14	0.000	0	-0.81	0.034289	0.936

[1]Seq: Sequencing platform used.

[2]NA: information not available.

[3]GW: gestational weeks.
