# Supplementary material for: Development of a comprehensive noninvasive prenatal test
Source: Genet Mol Biol. 2018 Jul 16;41(3):545–54. doi: 10.1590/1678-4685-GMB-2017-0177 (PMC6136382; doi:10.1590/1678-4685-GMB-2017-0177)
Supplement: Supplementary file 5 [file 1415-4757-GMB-1678-4685-GMB-2017-0177-suppl8.pdf]

Supplementary Material to "Development of a comprehensive noninvasive prenatal test"

Table S4 - Summary of non-pregnant individuals.

Sample	Relationship	Seq[1]	Trisomy	Gender	Mean bam coverage (X)	Mean SNP number	Expected FF	Fitted FF
F9966-1	Child	MiSeq	T21	Female	198.6	3866	0	0
F9966-2	Mother	MiSeq	Not-T21	Female	243	4589	0	0
F9219-1	Child	MiSeq	Not-T21	Female	186.8	5413	0	0
F9219-2	Mother	MiSeq	Not-T21	Female	156.6	4997	0	0
F10801-1	Child	HiSeq	Not-T21	Female	722.1	4105	0	0.01
F10801-2	Mother	HiSeq	Not-T21	Female	504.1	4171	0	0.02
F10114-1	Child	MiSeq	T21	Female	184.5	3867	0	0
F10114-2	Mother	MiSeq	Not-T21	Female	261.4	4445	0	0
C26449	Child	MiSeq	Not-T21	Male	223.4	4180	0	0.01
C26450	Mother	MiSeq	Not-T21	Female	265.3	4578	0	0
C25147	Child	MiSeq	Not-T21	Male	215	4364	0	0.01
C25148	Mother	MiSeq	Not-T21	Female	173	3902	0	0.01
C23944	Child	MiSeq	Not-T21	Male	92.85	2573	0	0.01
C23945	Mother	MiSeq	Not-T21	Female	84.55	1873	0	0.01
C20598	Child	MiSeq	Not-T21	Male	209.7	4204	0	0.01
C20672	Mother	MiSeq	Not-T21	Female	145.7	3437	0	0.04

[1]Seq: Sequencing platform used.
