# Supplementary material for: Development of a comprehensive noninvasive prenatal test
Source: Genet Mol Biol. 2018 Jul 16;41(3):545–54. doi: 10.1590/1678-4685-GMB-2017-0177 (PMC6136382; doi:10.1590/1678-4685-GMB-2017-0177)
Supplement: Supplementary file 6 [file 1415-4757-GMB-1678-4685-GMB-2017-0177-suppl1.pdf]

Supplementary Material to "Development of a comprehensive noninvasive prenatal test"

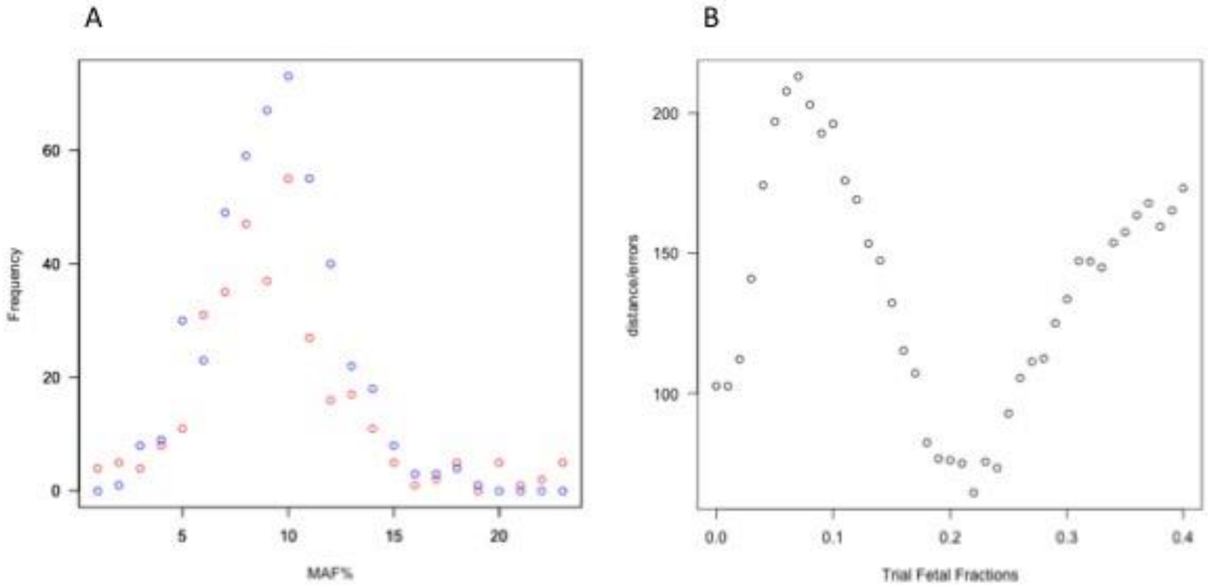

Figure S1 - Fit procedure for FF estimation. A) Distribution of MAF values for both test and simulated samples; Red: test sample; Blue: fitted simulated sample (most similar to the test sample). B) Fitting of the test sample to the closest simulated samples.
