# Supplementary material for: Development of a comprehensive noninvasive prenatal test
Source: Genet Mol Biol. 2018 Jul 16;41(3):545–54. doi: 10.1590/1678-4685-GMB-2017-0177 (PMC6136382; doi:10.1590/1678-4685-GMB-2017-0177)
Supplement: Supplementary file 7 [file 1415-4757-GMB-1678-4685-GMB-2017-0177-suppl2.pdf]

Supplementary Material to "Development of a comprehensive noninvasive prenatal test"

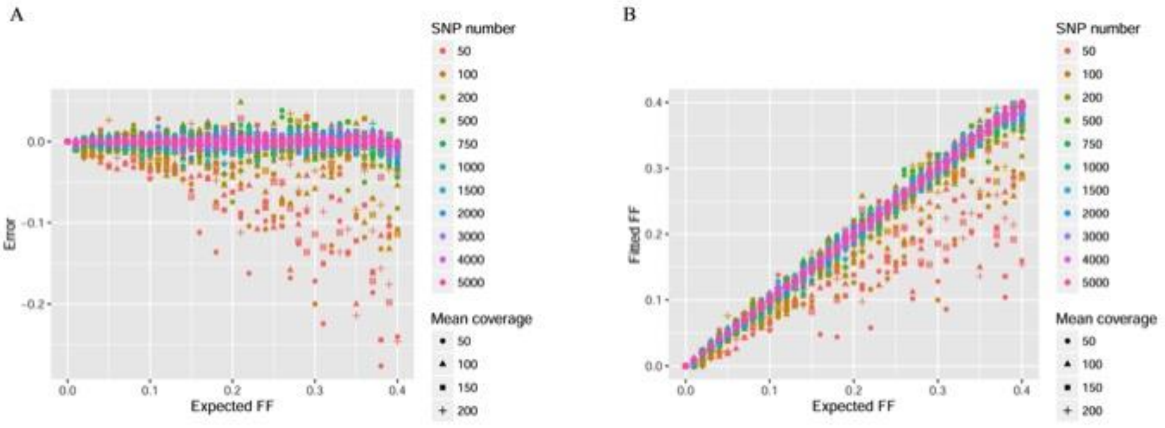

Figure S2 - Evaluation of the model according to different fetal fractions (FF). A) Evaluation of the fitting according to different mean coverage and SNP number. B) Error (Fitted - Expected) according to different mean coverage and SNP number. Shape and color incorporate both mean coverage and SNP number values, respectively.
