# Supplementary material for: Development of a comprehensive noninvasive prenatal test
Source: Genet Mol Biol. 2018 Jul 16;41(3):545–54. doi: 10.1590/1678-4685-GMB-2017-0177 (PMC6136382; doi:10.1590/1678-4685-GMB-2017-0177)
Supplement: Supplementary file 8 [file 1415-4757-GMB-1678-4685-GMB-2017-0177-suppl3.pdf]

Supplementary Material to "Development of a comprehensive noninvasive prenatal test"

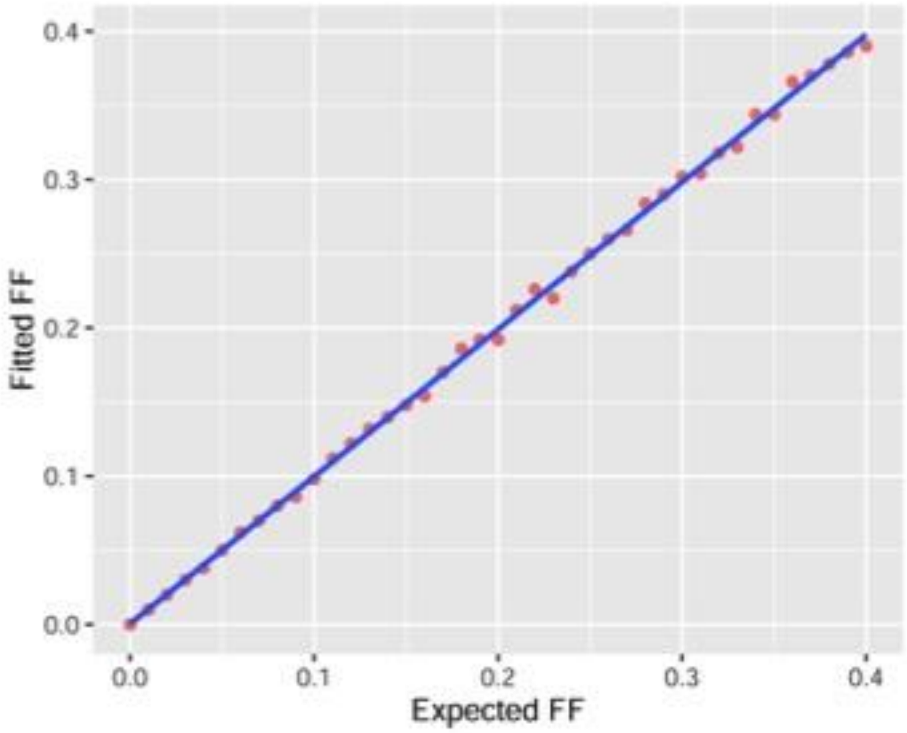

Figure S3 - Evaluation of the fitting for mean coverage value = 150X and SNP numbers = 2000. Shape and color incorporate both mean coverage and SNP number values, respectively
