# Supplementary material for: Development of a comprehensive noninvasive prenatal test
Source: Genet Mol Biol. 2018 Jul 16;41(3):545–54. doi: 10.1590/1678-4685-GMB-2017-0177 (PMC6136382; doi:10.1590/1678-4685-GMB-2017-0177)
Supplement: Supplementary file 9 [file 1415-4757-GMB-1678-4685-GMB-2017-0177-suppl9.pdf]

Supplementary Material to "Development of a comprehensive noninvasive prenatal test"

Table S5 - Summary of mock samples.

Sample[1]	Seq[2]	Trisomy	Fetal sex	Mean bam coverage (X)	Mean SNP number	Expected FF[3]	Fitted FF	Stdev FF	ChrY prop	ChrY Z-score	Chr21 prop	Chr21 Z-score
F9966_fetal4	MiSeq	T21	Female	254.6	4635	0.08	0.07	0.006	0	-0.81	0.035003	3.05
F9966_fetal5	MiSeq	T21	Female	257.8	4652	0.09	0.08	0	0	-0.81	0.035300	3.93
F9966_fetal10	MiSeq	T21	Female	272.5	4722	0.16	0.16	0	0	-0.81	0.036575	7.71
F9966_fetal15	MiSeq	T21	Female	285.4	4754	0.22	0.22	0.006	0	-0.81	0.037542	10.58
F9966_fetal20	MiSeq	T21	Female	296.6	4791	0.27	0.28	0	0	-0.81	0.038480	13.36
F9219_fetal4	MiSeq	Not-T21	Female	163.4	5197	0.09	0.09	0.01	0	-0.81	0.033722	-0.74
F9219_fetal5	MiSeq	Not-T21	Female	166.1	5227	0.11	0.11	0.006	0	-0.81	0.033679	-0.87
F9219_f	MiSeq	Not-	Female	177.8	5370	0.19	0.18	0.006	0	-0.81	0.0336	-0.85

**Sample[1]**	**Seq[2]**	**Trisomy**	**Fetal sex**	**Mean bam coverage (X)**	**Mean SNP number**	**Expected FF[3]**	**Fitted FF**	**Stdev FF**	**ChrY prop**	**ChrY Z-score**	**Chr21 prop**	**Chr21 Z-score**
etal10		T21									86	
F9219_fetal15	MiSeq	Not-T21	Female	187.6	5459	0.25	0.23	0.015	0	-0.81	0.033664	-0.92
F9219_fetal20	MiSeq	Not-T21	Female	195.8	5529	0.3	0.3	0.006	0	-0.81	0.033623	-1.04
F10801_fetal4	HiSeq	Not-T21	Female	330.4	3730	0.078	0.07	0.006	0	-1.06	0.034090	1.14
F10801_fetal5	HiSeq	Not-T21	Female	335.3	3737	0.094	0.09	0	0	-1.06	0.034069	1.05
F10801_fetal10	HiSeq	Not-T21	Female	357.9	3764	0.168	0.16	0	0	-1.06	0.034044	0.95
F10801_fetal15	HiSeq	Not-T21	Female	378.3	3790	0.23	0.22	0.011	0	-1.06	0.034062	1.03
F10801_fetal20	HiSeq	Not-T21	Female	396.6	3817	0.284	0.27	0.011	0	-1.06	0.034113	1.23
F10114_fetal4	MiSeq	T21	Female	269.8	4494	0.05	0.04	0	1.14E-06	1.62	0.034729	2.24
F10114_fetal5	MiSeq	T21	Female	271	4503	0.06	0.04	0.012	1.14E-06	1.62	0.034926	2.82
F10114_	MiSeq	T21	Female	275.8	4526	0.12	0.12	0	1.12E-	1.58	0.0357	5.37

Sample[1]	Seq[2]	Trisomy	Fetal sex	Mean bam coverage (X)	Mean SNP number	Expected FF[3]	Fitted FF	Stdev FF	ChrY prop	ChrY Z-score	Chr21 prop	Chr21 Z-score
fetal10									06		84	
F10114_ fetal15	MiSeq	T21	Female	279.3	4557	0.16	0.17	0.006	1.10E-06	1.53	0.036566	7.69
F10114_ fetal20	MiSeq	T21	Female	281.8	4566	0.21	0.22	0.01	1.09E-06	1.51	0.037233	9.66
C26449 _fetal4	MiSeq	Not-T21	Male	278.5	4640	0.07	0.06	0.01	3.87E-05	81.74	0.034290	0.94
C26449 _fetal5	MiSeq	Not-T21	Male	281.2	4654	0.08	0.08	0.006	4.75E-05	100.51	0.034342	1.09
C26449 _fetal10	MiSeq	Not-T21	Male	293.8	4723	0.15	0.14	0.006	8.71E-05	184.98	0.034405	1.28
C26449 _fetal15	MiSeq	Not-T21	Male	305	4775	0.21	0.19	0.006	0.00012418	264.09	0.034445	1.40
C26449 _fetal20	MiSeq	Not-T21	Male	314.7	4801	0.25	0.25	0	0.00015708	334.25	0.034523	1.63
C25147 _fetal4	MiSeq	Not-T21	Male	184.8	4063	0.09	0.09	0.006	6.01E-05	127.38	0.033624	-1.03
C25147 _fetal5	MiSeq	Not-T21	Male	187.5	4096	0.1	0.1	0.006	6.70E-05	142.10	0.033653	-0.95
C25147	MiSeq	Not-	Male	200	4220	0.18	0.18	0.006	0.0001	251.04	0.0337	-0.55

**Sample[1]**	**Seq[2]**	**Trisomy**	**Fetal sex**	**Mean bam coverage (X)**	**Mean SNP number**	**Expected FF[3]**	**Fitted FF**	**Stdev FF**	**ChrY prop**	**ChrY Z-score**	**Chr21 prop**	**Chr21 Z-score**
_fetal10		T21							1807		88	
C25147 _fetal15	MiSeq	Not-T21	Male	211	4325	0.24	0.25	0	0.00016716	355.76	0.033823	-0.44
C25147 _fetal20	MiSeq	Not-T21	Male	220.7	4407	0.29	0.29	0.01	0.00019371	412.39	0.033886	-0.26
C23944 _fetal4	MiSeq	Not-T21	Male	93.79	2575	0.12	0.12	0.006	3.70E-05	78.11	0.033904	-0.20
C23944 _fetal5	MiSeq	Not-T21	Male	106	3226	0.22	0.23	0.012	8.36E-05	177.51	0.033669	-0.90
C23944 _fetal10	MiSeq	Not-T21	Male	120.7	3940	0.31	0.32	0.006	0.00013173	280.18	0.033574	-1.18
C23944 _fetal15	MiSeq	Not-T21	Male	107.6	3408	0.25	0.26	0.006	7.26E-05	154.05	0.033761	-0.63
C23944 _fetal20	MiSeq	Not-T21	Male	111.3	3613	0.28	0.29	0	8.06E-05	171.11	0.033776	-0.58
C20598 _fetal4	MiSeq	Not-T21	Male	162.4	3766	0.14	0.12	0.006	6.73E-05	142.74	0.033758	-0.64
C20598 _fetal5	MiSeq	Not-T21	Male	166.4	3841	0.17	0.14	0.01	7.60E-05	161.30	0.033788	-0.55
C20598	MiSeq	Not-	Male	185.2	4101	0.26	0.27	0.006	0.0001	319.95	0.0339	-0.13

**Sample[1]**	**Seq[2]**	**Trisomy**	**Fetal sex**	**Mean bam coverage (X)**	**Mean SNP number**	**Expected FF[3]**	**Fitted FF**	**Stdev FF**	**ChrY prop**	**ChrY Z-score**	**Chr21 prop**	**Chr21 Z-score**
_fetal10		T21							5037		30	
C20598_fetal15	MiSeq	Not-T21	Male	201.7	4289	0.33	0.34	0.021	0.00020384	433.99	0.033862	-0.33
C20598_fetal20	MiSeq	Not-T21	Male	216.3	4439	0.38	0.39	0.015	0.00023816	507.21	0.033883	-0.27

[1]Sample name_fetal XX, where XX indicates the original fastq proportion admixture (in percentage).

[2]Seq: Sequencing platform used.

[3]Calculated by fetal-specific alleles using maternal genotype information.
